# 677 The Trauma-Informed Burn Center Care Team: A Toolkit for Practitioners

**DOI:** 10.1093/jbcr/iraf019.306

**Published:** 2025-04-01

**Authors:** Sydney Gudvangen, Lisa Kittleson

**Affiliations:** University of Minnesota, Regions Hospital Burn Center; University of Minnesota, Regions Hospital Burn Center

## Abstract

**Introduction:**

Trauma and mental illness can both precede and follow a burn injury, creating specialized mental health needs. While there is extensive research on the benefits of a trauma-informed organization, few studies provide information beyond the general need for trauma-informed care (TIC) in burns. This program development project aimed to identify and address the gap in burn-specific TIC to enhance practitioner knowledge and competence in using TIC at the bedside.

**Methods:**

A descriptive, mixed-methods needs assessment was used to examine pre-education TIC knowledge, opinions, and competence. The Trauma-Informed Care Provider Survey (n=37) analyzed knowledge, opinions favorable to TIC, self-rated competence, recent uses of TIC strategies, and perceived barriers. Six focus groups (n=21) were conducted to analyze the unit’s perceptions, unique cultural elements, and educational needs. Focus groups were recorded and audio-transcribed verbatim, then coded to generate descriptive commonalities. All data were compiled and analyzed to create a toolkit, which included evidence-based knowledge about trauma, signs and symptoms of trauma, methods within burn care that may impact trauma responses, descriptions of TIC, and specific TIC tools and strategies to be used at the bedside.

**Results:**

Pre-toolkit results found a lack of education and training in TIC among the care team. While approximately 60% of survey respondents identified correct responses on knowledge and 82% had favorable opinions of TIC, self-rated competence was approximately 50%. Focus group descriptive commonalities identified (1) barriers such as time constraints and discomfort, (2) current use of TIC without recognition under a TIC framework, (3) presence of practitioner secondary trauma, and (4) areas for improvement, including reducing traumatic aspects of burn care, using normalization and validation as helpful TIC tools, addressing how preconceived notions impact patient cares, and recognizing how language can influence patient responses. Findings also indicated a need for efficient yet quick methods to incorporate TIC at the bedside due to task-heavy and chaotic working environments. Following completion and delivery of the toolkit, stakeholder feedback identified methods for quick study, sustainability, and education on the ‘why’ behind TIC in burns as most beneficial in increasing knowledge, awareness, and use of TIC in bedside practice.

**Conclusions:**

Analyzing the TIC gaps within a regional burn hospital identified needs that influenced creation of a burn-specific, easy-to-use, and sustainable TIC toolkit. Incorporating the burn center’s unique cultural needs may enhance uptake of TIC knowledge and use.

**Applicability of Research to Practice:**

Creation of a burn-specific TIC toolkit has the potential to increase knowledge and competence among practitioners in identifying and managing trauma responses that may occur with burn survivors.

**Funding for the Study:**

N/A

